# The influence of M-CSF on fracture healing in a mouse model

**DOI:** 10.1038/s41598-021-01673-w

**Published:** 2021-11-16

**Authors:** Julia Starlinger, Kambiz Sarahrudi, Mathias Kecht, Florian Koerbler, Peter Pietschmann, Seyedhossein Aharinejad

**Affiliations:** 1 Department of Orthopedics and Trauma-Surgery, Medical University of Vienna, Vienna, Austria; 2 Department of Orthopedic Surgery, Mayo Clinic, Rochester, MN USA; 3 Department of Trauma Surgery, Landesklinikum Wiener Neustadt, Corvinusring 3-5, 2700 Wiener Neustadt, Austria; 4 Department of Orthopaedics and Trauma Surgery, Landesklinikum Baden Mödling, Baden, Austria; 5 Institute of Pathophysiology and Allergy Research, Center for Pathophysiology, Infectiology and Immunology, Medical University of Vienna, Vienna, Austria; 6 Department of Paediatrics and Adolescent Medicine, Villach Regional Hospital, Villach, Austria; 7 Unaffiliated, Vienna, Austria

**Keywords:** Preclinical research, Bone

## Abstract

Macrophage colony-stimulating factor 1 (M-CSF) is known to play a critical role during fracture repair e.g. by recruiting stem cells to the fracture site and impacting hard callus formation by stimulating osteoclastogenesis. The aim of this experiment was to study the impact of systemic M-CSF application and its effect on bony healing in a mouse model of femoral osteotomy. Doing so, we studied 61 wild type (wt) mice (18-week-old female C57BL/6) which were divided into three groups: (1) femoral osteotomy, (2) femoral osteotomy + stabilization with external fixator and (3) femoral osteotomy + stabilization with external fixator + systemic M-CSF application. Further, 12 op/op mice underwent femoral osteotomy and served as proof of concept. After being sacrificed at 28 days bony bridging was evaluated ex vivo with µCT, histological and biomechanical testing. Systemic M-CSF application impacted osteoclasts numbers, which were almost as low as found in op/op mice. Regarding callus size, the application of M-CSF in wt mice resulted in significantly larger calluses compared to wt mice without systemic M-CSF treatment. We further observed an anabolic effect of M-CSF application resulting in increased trabecular thickness compared to wt animals without additional M-CSF application. Systemic M-CSF application did not alter biomechanical properties in WT mice. The impact of M-CSF application in a mouse model of femoral osteotomy was oppositional to what we were expecting. While M-CSF application had a distinct anabolic effect on callus size as well as trabecular thickness, this on bottom line did not improve biomechanical properties. We hypothesize that in addition to the well-recognized negative effects of M-CSF on osteoclast numbers this seems to further downstream cause a lack of feedback on osteoblasts. Ultimately, continuous M-CSF application in the absence of co-stimulatory signals (e.g. RANKL) might overstimulate the hematopoietic linage in favor of tissue macrophages instead of osteoclasts.

## Introduction

Fracture repair requires equilibrium of cellular and molecular mechanisms aiming towards bone formation especially osteoblasts and osteoclasts and their respective effector molecules^1,2^. Osteoblasts, which derive from mesenchymal stem cells (MSC), are responsible for the production of bone extracellular matrix and are able to mineralize into bone matrix. On the contrary, osteoclasts originate from the monocyte/macrophage/hematopoietic lineage and are responsible for bone resorption^3^. Osteoblasts produce soluble Macrophage colony-stimulating factor 1 (sM-CSF) and membrane-bound M-CSF (mM-CSF) which binds to the colony stimulating factor 1 (CSF) receptor of pre-osteoclasts stimulating osteoclastogenesis^4^. Thus, M-CSF regulates the resorptive activity of tissue specific macrophages and osteoclasts^5,6^. Ultimately, M-CSF seems to play a crucial role in osteoblast-mediated osteoclast production within the bone^7^. Nevertheless, current evidence is conflicting whether exogenous M-CSF acts anabolic or negatively impacts bone formation: some authors suggest that M-CSF signaling restricts osteoclast formation and thereby protects bone^8–12^ whilst others suggest M-CSF to be anabolic by promoting osteoclastogenesis^13,14^.

Besides M-CSF the co-stimulatory molecules Receptor activator of nuclear factor kappa-Β ligand (RANKL) and osteoprotegerin (OPG) contribute to the M-CSF/RANKL/OPG axis, which is key for bidirectional signaling between osteoblasts and osteoclasts. The crucial role of M-CSF has been documented in in vitro studies, as human osteoclast precursor cells treated with M-CSF increased their proliferation irrespective of additional RANKL application. Hence, the authors suggested M-CSF signaling to be an independent mediator in an effort to target osteoclastogenesis, which is in line with the current literature^15,16^.

The substantial role of M-CSF in bone resorption has been nicely illustrated in vivo in the context of op/op mice. op/op mice are homozygous for the recessive osteopetrosis spontaneous mutation, causing a complete absence of a functional M-CSF. Thus, op/op mice present with obliterated osteoclast numbers, macrophages as well as monocytes resulting in generalized osteopetrosis. Interestingly, treatment with M-CSF in op/op mice has been reported to enhance the local angiogenesis and support normalization of the macrophage-, osteoclast- and osteoblast population. Doing so, op/op mice were reported to be partially or completely cured after treatment with systemic M-CSF treatment^17–20^.

However, only few data on the role of M-CSF with regard to fracture healing exist so far. We documented the significance of M-CSF during human fracture healing in 113 patients, who were treated for long bone fractures. M-CSF levels were found to be significantly elevated in the fracture hematoma at the time of injury as well as in patient’s serum over the entire observation period with peak levels of M-CSF at week 1 and 2 after injury emphasizing the active involvement of this molecule in the fracture healing process from the early inflammation phase on. Serum concentration of M-CSF stayed elevated during the entire healing period indicating its importance during the fracture healing process^21^.

We then transferred these observations into a rabbit model to study the impact of systemic M-CSF application in of critical size bone defects treated with conventional plating^22^. Doing so, M-CSF application increased bone mass by 2.6 times at 4 weeks and 2.1 times at 8 weeks after operation compared to controls. Moreover, animals treated with M-CSF displayed higher osteoclasts numbers compared to controls linking M-CSF application and osteoclast numbers in vitro.

The very opposite was studied by Shantz et al., who treated tibial shaft fractures in mice with a PLX3397, which is a CSF-R1 inhibitor. Doing so, the authors found decreased numbers of macrophages in the fracture callus as well as distinct differences with respect to the animals’ age. Interestingly, PLX3397 treatment caused larger calluses and increased bone formation in elderly animals^23^. Alexander et al. studied the impact of CSF-1 administration in a mouse tibial injury model and its impact on the number on macrophages. The authors observed a significant increase in the number of resident macrophages (i.e. osteomacs) at the site of injury, which was a hole drilled through the entire diameter of the proximal tibia that healed by intramembranous bone formation^8^.

Given the significance of M-CSF in the context of bony healing M-CSF might open a possible therapeutic window for anabolic treatment in an effort to support bony union in a murine fracture model. We hypothesized that systemic M-CSF application enhances endochondral fracture healing through the stimulation of osteoclastogenesis and reinforces the biomechanical properties in a mouse model of femoral osteotomy.

## Material and methods

### Animal surgery and M-CSF treatment

This animal project was approved by the Animal Ethics Committee of the Medical University of Vienna (TV 132/11). All animal care was carried out according to the Principles of Laboratory Animal Care (NIH publication No. 86-23, revised 1985) as well as in accordance to the ARRIVE guidlines. Eighteen weeks old female heterozygote osteopetrosis mice (op/op) (“B6C3Fe a/a-CSF1 op/J”; Research Institute for Laboratory Animal Breeding, Himberg, Austria) as well as corresponding 18-week-old female wild type (WT) mice (C57BL/6; Charles Rivers Laboratories) were obtained. Op/op mice are characterized by an inactivating mutation in the CSF-1 gene resulting in the absence of biologically active M-CSF. Consequently, op/op mice have impaired mononuclear phagocyte development characterized by a deficiency of osteoclasts resulting in severe osteopetrosis^24,25^.

Animals were anesthetized with 0.1 ml/10 g of a combination of ketamine 0.5 ml + 0.15 ml Rompun + 0.1 ml Dormicum in 5 ml NaCl which we administered subcutaneously. As for post-operative pain management animals received Buprenorphin 0.1 mg/kg s.c. at the end of the surgery and Piritramid 15 mg/250 ml in drinking water ad libitum until post-operative day 5.

All animals received an unilateral osteotomy on their right femur. A transverse osteotomy was created in the mid-diaphysis with a 0.66 mm Gigli saw. The osteotomy was stabilized with an external fixator (RIS, www.risystem.com). Twenty randomly assigned WT mice were implanted a subcutaneous, osmotic mini-pump (Alzet, Paolo Alto, CA), which was placed aseptically subcutaneously through a skin incision adjacent to the abdominal wall and was left in place upon sacrifice. The mini-pumps were used as continuous drug delivery system, delivering their content (2 × 10^6^ I.E. recombinant mouse M-CSF (Fa. Biomedica, Vienna, Austria) which was reconstituted in 100 μg/mL in sterile phosphate-buffered saline) at a rate of 24 mg/day over a period of 4 weeks. The remaining WT mice as well as the op/op mice did not receive systemic M-CSF treatment nor a sham-implantation of a pump.

**Figure 1:**
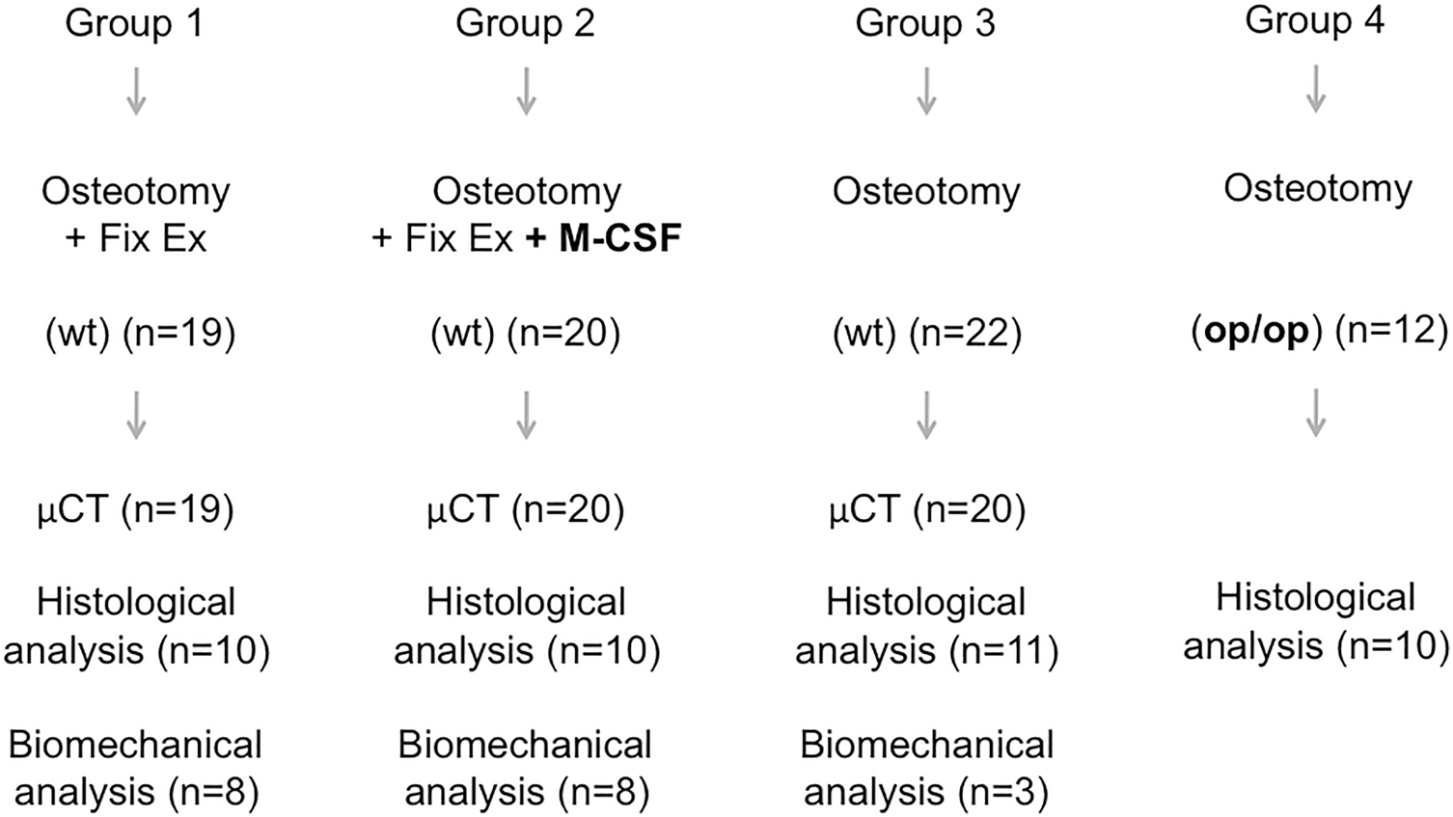
Design of study groups. *M-CSF* macrophage-colony stimulating factor, *wt* wildtype, *op/op* osteopetrosis knock out mice, *Fix Ex* external fixator, *st* stabilization.

Ultimately, we studied three respective groups of wt animals which were (1) treated with osteotomy and external fixator (“group 1”) (2) treated with osteotomy + external fixator + systemic M-CSF application (“group 2”) or were (3) treated with osteotomy only (“group 3”). Further we studied a group of twelve op/op mice, which were treated with osteotomy only and are further referred to as “group 4” (Fig. 1).

### Workup

After being sacrificed at 28 days fractures from all femurs were harvested for further analysis. After careful removal of the external fixator bony bridging was evaluated ex vivo by micro-computed tomography (µCT). Further, histological and biomechanical parameters were assessed.

### µCT

All femurs were scanned using µCT (µCT 35, Scanco Medical, Brüttisellen, Switzerland). The X-ray tube was operated at 70 kVp and 114lA with an integration time set to 300 ms. Scans were performed at an isotropic, nominal, and spatial resolution of 10 lm (high-resolution mode). A threshold of 220 was used. Parameters of interest were assessed, connectivity density (Conn.D.), bone volume/total volume (BV/TV), trabecular number (Tb.N), trabecular thickness (Tb.Th) and trabecular separation (Tb.Sp), respectively. The region of the former osteotomy gap was defined as the region of interest.

### Histology

**Figure 2:**
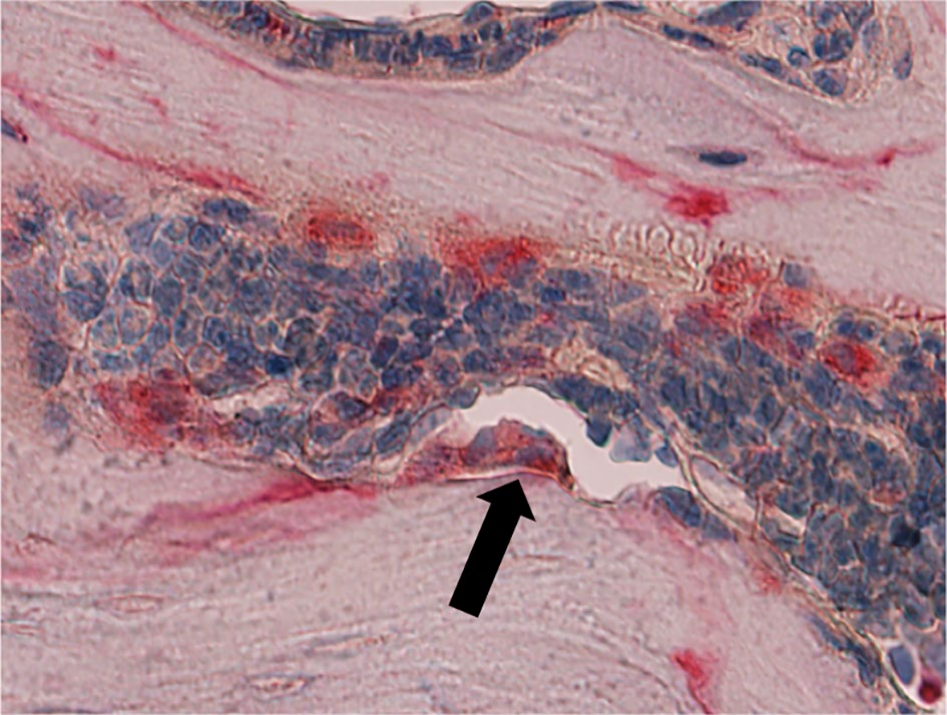
Representative TRAP staining of a wild type mouse with a femoral osteotomy stabilized with an external fixator. The arrow shows an osteoclast; original magnification: ×400 (source: Florian Körbler, diploma thesis, Medical University of Vienna).

The collected specimens were fixed in 4% formaldehyde decalcified in Tris–EDTA solution, dehydrated and embedded in paraffin. For histology, the paraffin specimens were cut into 4 μm serial longitudinal tissue sections and stained with hematoxylin and eosin. In order to determine osteoclast parameters, the sections were stained for tartrate resistant acid phosphatase (TRAP, Sigma) and counterstained with hemalum (Fig. 2). Histological sections were evaluated blinded by one observer (KF) using the OsteoMeasure system (OsteoMetrics, Decatur, Georgia, USA). The callus size represents the cross sectional area of a histologically representative sample. Osteoclasts were counted as multinucleated TRAP positive cells; we determined the number of osteoclasts per callus area and the osteoclast perimeter.

### Biomechanical testing

The specimens were subjected to a three-point bending test with a universal testing machine (Z050, Zwick GmbH & Co, Ulm, Germany) using a 20 N test load. The femora were placed at their lateral surface on the lower supports at a width of 8 mm in between. Before actual testing a small stabilizing preload (0.5 N) was applied at a speed of 0.155 mm/s. Fracture force to failure and modulus of elasticity were calculated from the load curves.

### Statistical analysis

Data are presented as the median ± interquartile range, comparisons were made using a Mann–Whitney-U-Test. Statistical analysis was performed using SPSS version 25.0 (SPSS, Inc., Chicago, IL, USA). P < 0.05 was considered to indicate a statistically significant difference.

## Results

### M-CSF application caused osteoclast numbers in WT mice drop to levels as low as found in op/op mice

**Figure 3:**
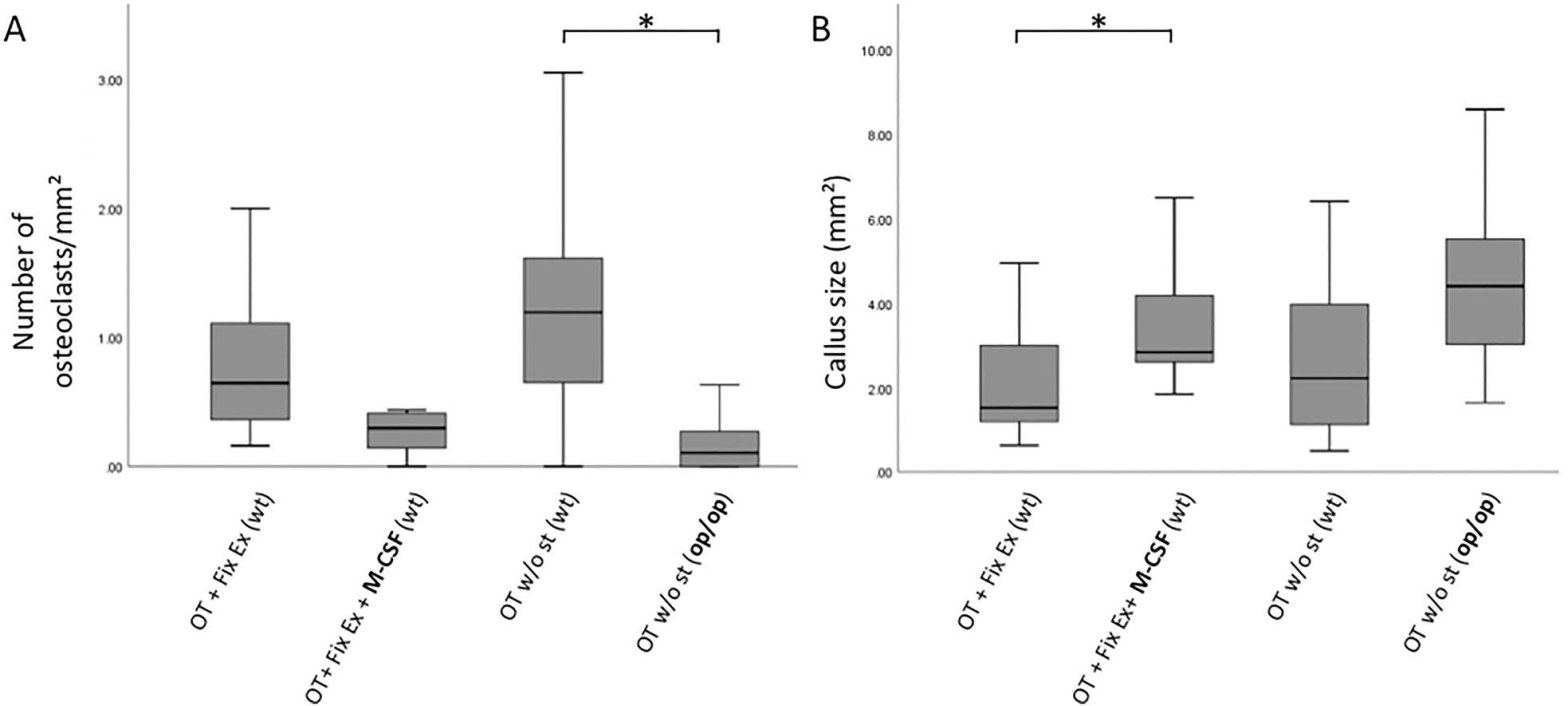
(**A**, **B**) Histologic analysis revealed op/op mice (group 4) displayed significantly less osteoclasts/mm^2^ compared to wt mice (group 3) (P = 0.001) (both groups without additional stabilization). M-CSF treatment had a striking effect on callus size compared to wt mice without M-CSF treatment (P = 0.043). *M-CSF* macrophage-colony stimulating factor, *wt* wildtype, *op/op* osteopetrosis knock out mice, *OT* osteotomy, *Fix Ex* external fixator, *st* stabilization.

Studying the impact of M-CSF on osteoclast numbers in a knock out model we found the lowest number of osteoclasts/mm^2^ in op/op mice, which were significantly lower than in the corresponding group of WT mice (median osteoclast number/mm^2^ in group 4: 0.1043 mm, IQR: 0.000–0.2909 vs. median osteoclast number/mm^2^ in group 3: 1.19 mm, IQR: 0.44–1.65, P = 0.001, Fig. 3A). Of note, osteoclasts in group 4 (op/op mice) showed decreased color intensity of their osteoclasts compared to WT mice.

Concomitantly, the pronounced difference in osteoclast numbers corresponded inversely to the size of the respective callus: therefore, op/op mice (group 4) tended to have the largest callus compared to WT mice (group 3, P = 0.118). This was an interesting observation regarding the relevance of M-CSF (Fig. 3A,B).

### M-CSF has a relevant impact on osteoclast numbers and callus size in WT mice

After we had documented that M-CSF is clinically relevant we reached out to study the effect of M-CSF application in the context of fracture healing in WT mice. Interestingly, studying WT mice treated with external fixator, which received M-CSF treatment (group 2) presented with less osteoclasts/mm^2^ yet larger callus compared to group 1 (no M-CSF) (median callus size group 1: 1.52 mm^2^, IQR: 1.15–3.0 vs. median callus size group 2: 2.84 mm^2^, IQR: 2.52–4.2, P = 0.043, Fig. 3B). This inversely corresponded to a pronounced difference in osteoclast numbers/mm^2^: group 2 (WT mice with external fixator and concomitant M-CSF therapy) showed lower numbers of osteoclasts/mm^2^ than WT mice in group 1 (external fixator without additional M-CSF therapy), this difference was found to be of borderline significance (P = 0.057). Despite the application of M-CSF, which was expected to increase the number of osteoclasts, the actual number of osteoclasts/mm^2^ in group 2 was almost as low as in group 4, i.e. the M-CSF depleted op/op mice.

### Anabolic effect on trabecular thickness by M-CSF application

**Figure 4:**
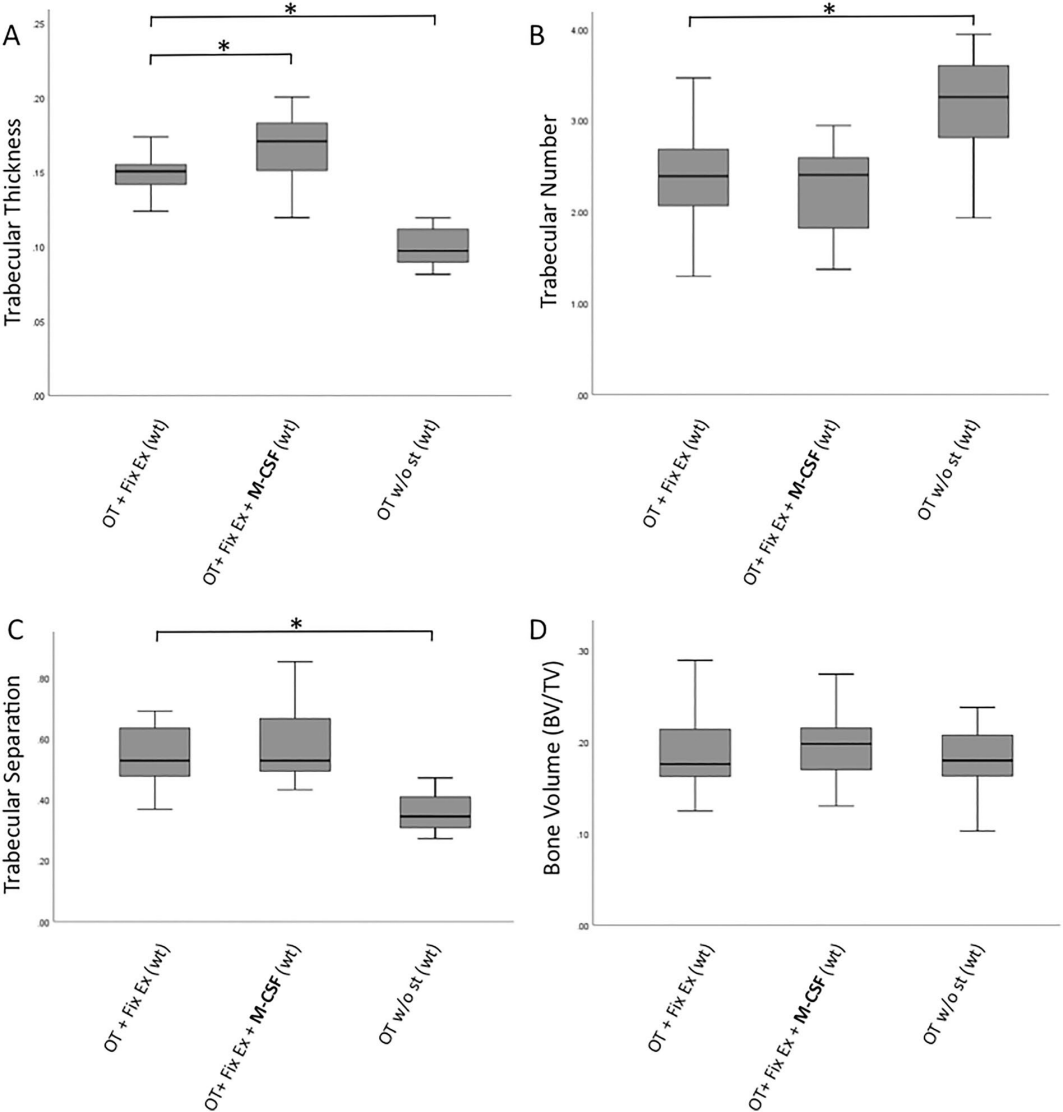
(**A**–**D**) Outcomes for trabecular bone microarchitecture (μCT): M-CSF treatment had a significant impact on fracture healing in mice treated with external fixator with respect to trabecular thickness (median trabecular thickness group 3: 0.0970, IQR: 0.0895–0.1120 vs. 0.1506, IQR: 0.1404–0.1587, P < 0.001). Interestingly, overall bone volume in terms of BV/TV was comparable between groups. *M-CSF* macrophage-colony stimulating factor, *wt* wildtype, *op/op* osteopetrosis knock out mice, *OT* osteotomy, *Fix Ex* external fixator, *st* stabilization.

While the number of osteoclasts/mm^2^ was decreased in WT mice treated with M-CSF, M-CSF application had a pronounced impact on trabecular thickness (Fig. 3A): M-CSF treated WT mice were found to have increased trabecular thickness compared to WT mice without additional M-CSF application (group 2), this difference was found to be statistically significant (median trabecular thickness in group 1: 0.1, IQR: 0.89–0.12 vs. median trabecular thickness in group 2: 0.13, IQR: 0.11–0.14, P = 0.006, Fig. 4A).

WT mice without stabilization showed the lowest values in terms of trabecular thickness compared to group 1 (median trabecular thickness in group 3: 0.1, IQR: 0.09–0.11 vs. median trabecular thickness in group 1: 0.15, IQR: 0.14–0.16, P < 0.001, Fig. 4A). Interestingly, mice in group 3 (wt mice without additional stabilization) presented the highest absolute number of trabeculae compared to WT mice treated with external fixator (group 2) (median trabecular number in group 3: 3.25, IQR: 2.73–3.62 vs. median trabecular number in group 1: 2.39, IQR: 1.99–2.70, P = 0.002, Fig. 4B).

At the same time WT mice without any stabilization (group 3) hat a significantly lower mean distance between trabeculae in terms of trabecular separation compared to mice with osteosynthesis with external fixator (group 2) (median trabecular separation in group 3: 0.35, IQR: 0.31–0.42 vs. median trabecular separation group 1: 0.53, IQR: 0.46–0.65, P < 0.001, Fig. 4C.) While the differences regarding trabecular thickness, trabecular number and trabecular separation were distinct in response to fracture stabilization as well as M-CSF treatment, the bone volume fraction in terms of BV/TV was comparable between wt mice irrespective of stabilization with external fixator or additional M-CSF application (Fig. 4D).

### M-CSF application did not affect bone strength in a clinical model

**Figure 5:**
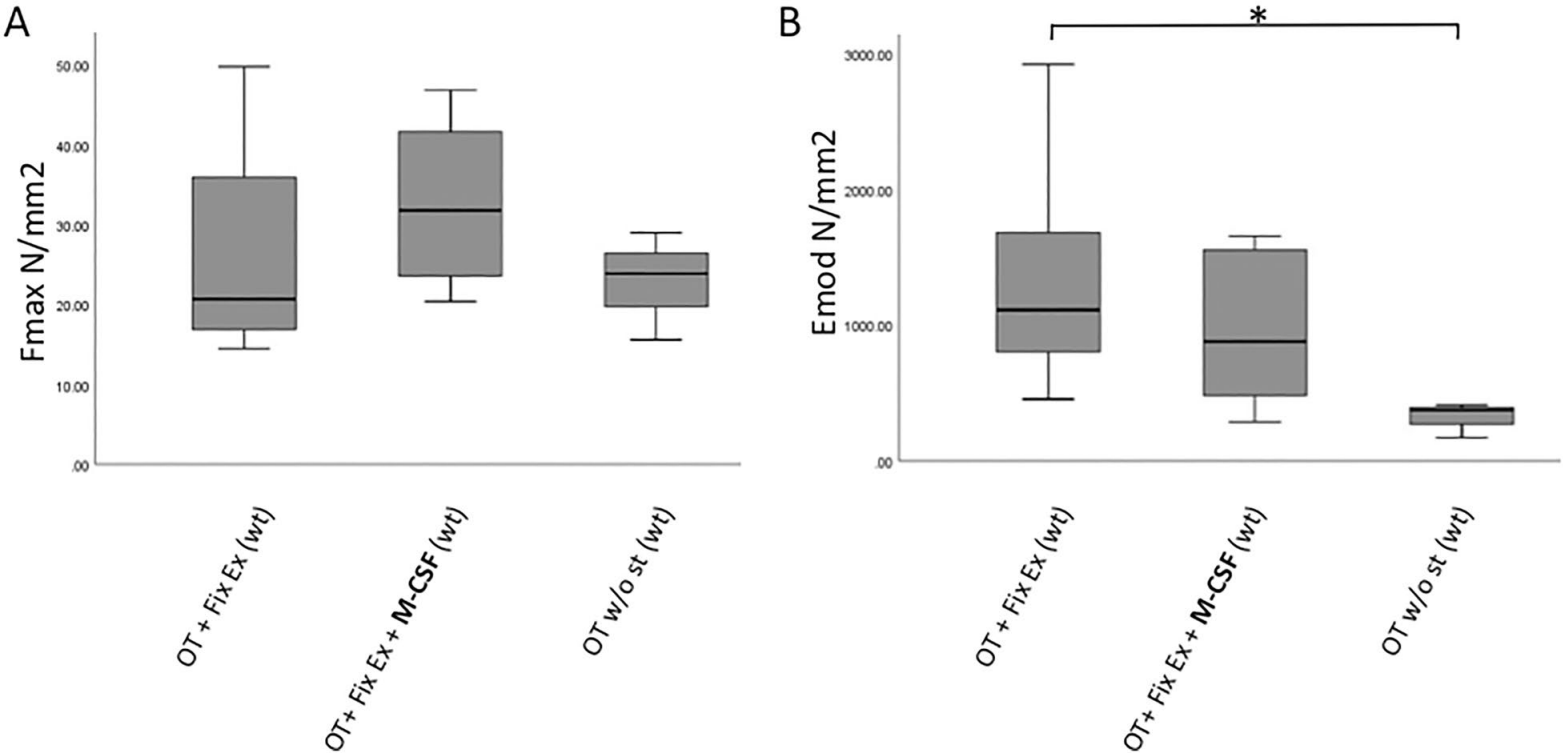
(**A**, **B**) Biomechanical analysis revealed a subtle increase in maximum force to failure associated with M-CSF application, but the difference was not found to be significant (P = 0.195). Ultimately, bony bridging was decreased in WT mice without fracture stabilization as reflected by significantly affected E modulus values. *M-CSF* macrophage-colony stimulating factor, *wt* wildtype, *op/op* osteopetrosis knock out mice, *OT* osteotomy, *Fix Ex* external fixator, *st* stabilization.

With respect to maximum force to failure we observed a trend towards increased values for WT mice treated with M-CSF compared to mice without additional M-CSF treatment, but the difference was not found to be statistically significant (group 1 vs. group 2, P = 0.195, Fig. 5A).

While we observed comparable values regarding maximum force to failure in mice with external fixation (± M-CSF treatment), WT mice without any stabilization of their femur osteotomy showed the lowest E modulus compared to those mice, which had additional fixation. This corresponds to an overall beneficial impact on fracture healing by fracture stabilization (median E modulus in group 3: 375, IQR: 173–375, median E modulus in group 1: 1112.5, IQR: 795.75–1761; P = 0.012, Fig. 5B). Correspondingly, maximum surface strain was highest in mice treated without stabilization.

## Discussion

The pro-anabolic role of M-CSF during fracture healing is well recognized, e.g. by M-CSF recruiting precursor cells to the fracture site, stimulating osteoclastogenesis, oc function and proliferation^26–30^. A complete absence of M-CSF as seen in op/op mice results in severe osteopetrosis, which is cured by iatrogenic M-CSF application and reconstitutes almost normal bone structure^24,25^. Given the powerful role of M-CSF we hypothesized, that systemic M-CSF application in a mouse model of femoral osteotomy might increase the osteoclast-osteoblast coupling as suggested by Kaur et al. and thereby stimulate osteoclastogenesis, increase bone turnover and ultimately enhance fracture healing in WT animals^31^.

Given the fact that mature osteoclasts contain M-CSF receptor transcripts^32–34^ we were curious to see the effect of systemic M-CSF application on osteoclast numbers. As expected, op/op mice displayed strikingly low osteoclast numbers compared to wt mice without stabilization, which showed highest osteoclast numbers/mm^2^ (Fig. 3A). While this finding served as a proof of concept, we were struck by the impact which systemic M-CSF application had on wt mice: while we expected M-CSF to stimulate the monocyte/macrophage/hematopoietic cell line resulting in high osteoclast numbers, M-CSF was not found to sufficiently induce osteoclast numbers in wt mice. On the contrary, osteoclast numbers/mm^2^ in wt mice treated with systemic M-CSF were almost as low as in our op/op mice. Given the existing paradox about the role of M-CSF in the context of fracture healing other factors like distinct timing of M-CSF application within the course of fracture healing should be evaluated as recently proposed by Raggatt et al. who studied the impact of M-CSF application at day five after surgery in macrophage Fas-induced transgenic mice^35^.

Further, systemic M-CSF application had a pronounced impact on callus size as well as bone microstructure: callus size in animals treated with M-CSF was significantly larger compared to WT animals without treatment (Fig. 3B). This is an interesting finding given the young age of the animals we used i.e. 18 weeks: M-CSF application provided for not only larger calluses but also significantly increased trabecular thickness. This is in sharp contrast to data published by Shantz et al. as well as Clark et al., who studied the effect of PLX3397. This drug is a M-CSF inhibitor which paradoxically resulted in increased bone formation compared to controls especially in old mice^36^. The age of the animals was 3 months (“young”) and 24 months (“old”). The authors showed that old mice (24 months) display delayed fracture healing with significantly less bone and more cartilage compared to young animals^23,26^. Further, the callus size was decreased in old animals as compared to young animals. Therefore, a potential age cut-off regarding the beneficial effects of PLX3397 should be evaluated, as well as for M-CSF. Given the impact of age Shantz et al. postulate that the inhibition of macrophages by blocking the M-CSF receptor c-fms might be a potential therapeutic target in elderly animals to enhance fracture healing in this specific subgroup^23^. Clearly, the significance of age in the context of osteoclast as well as macrophage activity needs more attention in an effort to clarify the impact on callus size and quality.

**Figure 6:**
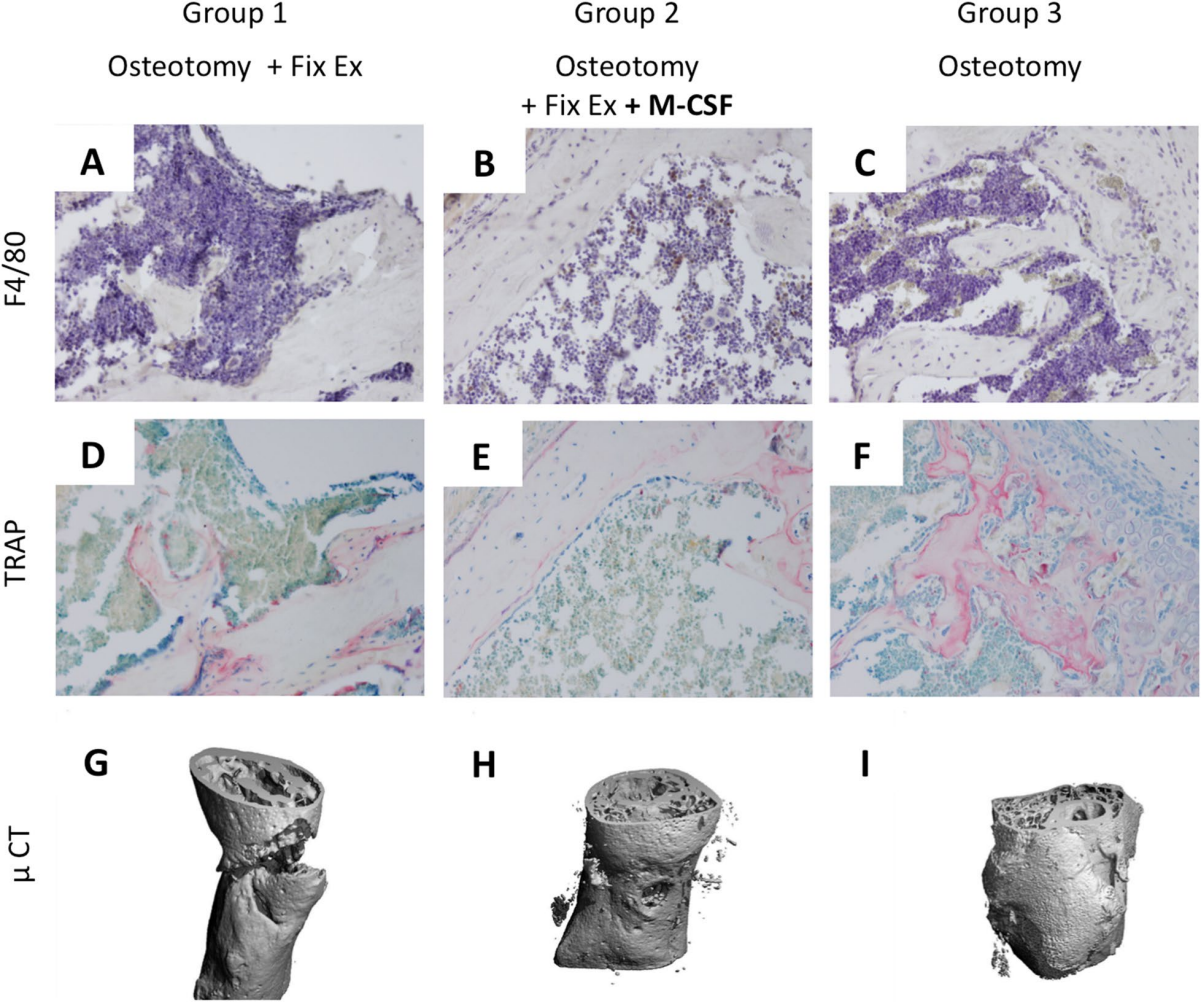
F4/80 staining (**A**–**C**) visualizing monocytes and TRAP staining (**D**–**F**) visualizing osteoclasts of exemplary slices of the three groups of wildtype mice are show here. Corresponding µCT pictures are shown in (**G**–**I**). (**A**) 6D and 6G correspond to mice treated with OT (ot) and external fixator (group 1). (**B**, **E**, **H**) represent mice treated with OT (osteotomy), external fixator and M-CSF (group 2). (**C**, **F**, **I**) represent mice treated with OT only (group 3). To visualize osteoclasts, the sections were stained for tartrate resistant acid phosphatase (TRAP, Sigma) and counterstained with toluidine blue. Monocytes were visualized using F4/80 antibodies (Invitrogen PA5-32399), then treated with HRP (Dako EnVision system, Dako A/S, Glostrup, Denmark) and ultimately counterstained with hemalum.

In our study M-CSF had a notable impact on fracture healing with respect to trabecular thickness although total bone volume (BV/TV) was not impacted. These results are in line with previously reported data on M-CSF administration and new bone formation in a rabbit model^37^. However, the fact that administration of M-CSF led to a reduction of the number of osteoclasts was unexpected. It is tempting to speculate that this finding might be caused by a stimulatory impact on the osteoblastic MSC niche i.e. a direct stimulatory effect of M-CSF on osteoblasts resulting in increased trabecular thickness. But to date only cells of the monocyte/macrophage/hematopoetic lineage are recognized to display a CSF-receptor, which makes a direct stimulatory effect of M-CSF on osteoblasts rather unlikely. Rather, the lack of osteoclasts in WT mice treated with systemic M-CSF is potentially responsible for missing inhibitory feedback mechanisms further downstream. Physiologically, regular numbers of osteoclasts would control and limit osteoblast activity. Accordingly, the missing feedback of osteoclasts mimics a pro-osteoblastic effect, which imitates a shift towards the MSC lineage. As shown in Fig. 6 the systemic application of M-CSF was associated with a higher number of F4/80 positive cells in the bone marrow when compared to mice that did not receive M-CSF. Ultimately, an additional pro-osteoblastic feedback mechanism cannot ultimately be ruled out. To this end, our findings are in sharp contrast to what we might have expected, which was an increased number of osteoclasts in those mice receiving systemic M-CSF therapy. Obviously, the microenvironment of bidirectional signaling is at risk in face of continuous and systemic M-CSF application. Lacking osteoclast derived coupling factors resulted in increased callus size as well as increased trabecular thickness but ultimately failed to increase biomechanical properties in our experiment.

**Figure 7:**
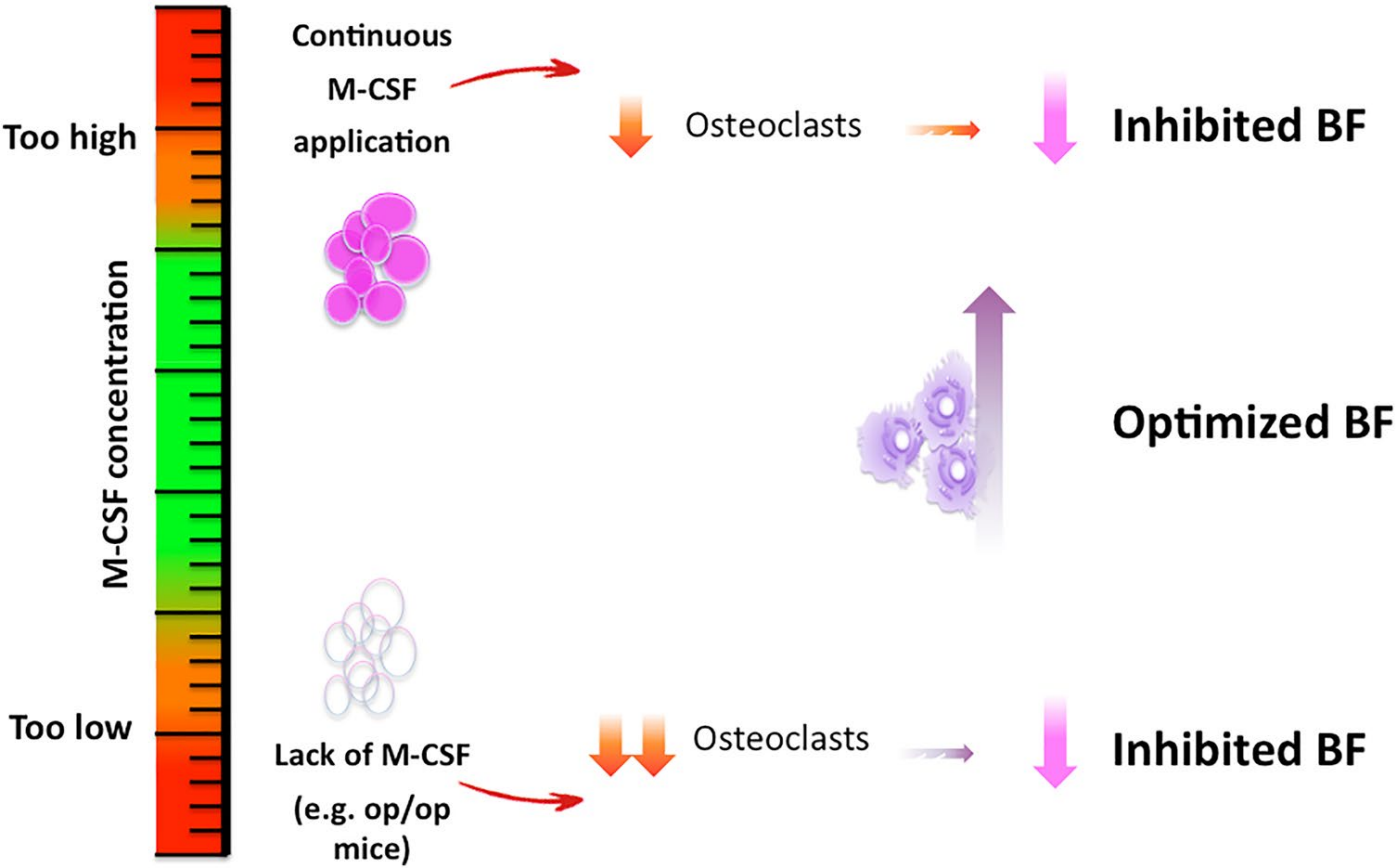
Systemic M-CSF concentration is crucial for it’s effect on osteoclasts and any downstream mechanism. *M-CSF* macrophage colony-stimulating factor, *op/op* osteopetrosis knock out mice, *BF* bone formation.

M-CSF expression by osteoblasts is required for pre-osteoclasts to differentiate into osteoclasts, but M-CSF on its own is obviously unable to enhance this process. While in vitro data suggests that M-CSF is able to stimulate osteoclast development irrespective of additional co-stimulatory signals, e.g. RANKL, this might not be the case in vivo. Most recent data suggest that continuous M-CSF application in the absence of co-stimulatory signals such as RANKL might overstimulate the hematopoietic linage in favor of tissue macrophages instead of osteoclasts. Accordingly, there might be a small therapeutic window of M-CSF concentration where osteoclast activity is just perfectly orchestrated or optimized (Fig. 7). But beyond this concentration, osteoclastogenesis might be out of balance and precursor cells of the monocyte/macrophage/hematopoietic cell line might be rather aiming towards tissue macrophages, which are then likely to leave the bone.

The tight coupling between bone formation and resorption represents a thin line when aiming to influence this process. The suppression of osteoclasts might have had a positive impact on the trabecular thickness while the net effect regarding bone volume (BVTV) was zero. Ultimately, a co-stimulation of M-CSF together with RANKL might be required to ultimately support cell differentiation into osteoclasts instead of tissue macrophages or osteomacs.

The following limitations of our analyses need to be acknowledged. In an effort to demonstrate the significant role of M-CSF, we initially aimed to treat op/op mice with M-CSF to overcome their osteoclast depletion. Despite a meticulous surgical technique we failed to mount external fixators given the animals severe osteopetrosis. This forced us to modify the study design to which is presented here instead of comparing three groups that were all treated with external fixation (Fig. 1). Of note, control animals in our study did not receive any vehicle treatment or sham-implantation of a pump. While the effect is most likely minor, this needs to be considered as limitation when interpreting our results. Further, different mouse strains have been reported to significantly differ in terms of fracture healing. Investigated B6C3Fe a/a-CSF1 mice have been generated on the background of multiple mouse stains, including C57BL/6 but also C3H mice, which have been demonstrated to differ in fracture healing dynamics^38^. As we used C57BL/6 mice as control, potential effects of the background mouse stains in B6C3Fe a/a-CSF1 mice need to be taken into account, as they could potentially have affected fracture healing as well. Another limitation is the fact that our assessment focused exclusively on post-operative day 28 instead of assessing multiple time points which would provide a more detailed picture of e.g. oc numbers during the course of fracture healing. In future studies the time course should be assessed in more detail by e.g. assessment of monocyte recruitment to the fracture site via F4/80 staining at pre-defined time points.

Ultimately, our results contribute to the deeper understanding of the crucial importance of osteoclasts in the setting of op/op mice and potential obstacles that are associated with using op/op mice in models of fracture healing. Concomitantly our results stress the crucially important fine-tuning between bone resorption by osteoclasts and bone formation by osteoblasts in an effort to support fracture healing in the future.
